# Power, Control, and Optimization

**DOI:** 10.1155/2015/964581

**Published:** 2015-02-19

**Authors:** Pandian Vasant, Gerhard-Wilhelm Weber, Nader Barsoum, Vo Ngoc Dieu

**Affiliations:** ^1^Department of Fundamental and Applied Sciences, Universiti Teknologi Petronas, Malaysia; ^2^Institute of Applied Mathematics, METU, Turkey; ^3^School of Engineering and Science, Curtin University, Sarawak, Malaysia; ^4^Department of Power Systems, University of Technology, Ho Chi Minh City, Vietnam

This special issue of this journal is devoted to the* 7th Global Conference on Power, Control, and Optimization* (*PCO'2013*) (http://ifors.org/web/seventh-global-conference-on-power-control-and-optimization-pco-2013/), which was held on August 25–27, 2013, in Prague, Czech Republic. Best papers of this conference were covered.* PCO'2013* attracted participants from many countries and from across the* PCO* community in Europe and all over the world, for a meeting of vivid exchanges and lively debates. The* PCO'2013* conference provided an excellent floor for investigators and practitioners to promote their newest advances in power, control, and optimization to the wider community of scientists and practitioners, for identifying research challenges for their fields, as well as promising research developments in theory, methods, and applications, and for fostering given and creating new interactions with colleagues from related research areas of modern* PCO* areas and their emerging applications.

Applications of optimization techniques are well known in the research areas of power systems, control systems, and computer science. The current trends on application of metaheuristics techniques are very popular among the researchers in the areas of power systems, control systems, and computer science across the globe. In this regard, the* PCO* global conference organized a successful conference in the research areas of power systems, control systems, and optimization in Prague, the capital of Czech Republic. This special issue focuses on best and high quality selected papers which were presented at the PCO global conference. Well-known and new methodologies and techniques of optimization were used to solve some of the complicated and hard problems in the areas of power systems, control systems, and optimization.

This special issue was open for interested authors in all power, control, and optimization areas, and they were welcome to submit their recent findings and best results. Both* PCO'2013* and our special issue focused on new development and contribution of the current research to the body of the knowledge in the area of* PCO*. The topics included are (i) optimal power; (ii) optimal control; (iii) optimization; (iv) cams, gears, wheels, and valve optimization; (v) android phone, mobile, WiMax, and wireless communications; (vi) smart grid, microgrid, distribution, and chaotic systems; (vii) scheduling and assignment problems; (viii) healthcare, bioinformatics, and signal processing; and (ix) future energy planning and the environment.

The subjects of power, control, and pptimization as elaborated in our special issue are so closely related to the emerging questions and challenges on energy and electricity, from engineering, applied mathematics, operational research, computer science and data mining, education, and social sciences, both quantitatively and qualitatively, and they reach out very far and, eventually, to the living conditions of people on earth.

